# Targeting Key Risk Factors for Cardiovascular Disease in At-Risk Individuals: Developing a Digital, Personalized, and Real-Time Intervention to Facilitate Smoking Cessation and Physical Activity

**DOI:** 10.2196/47730

**Published:** 2024-12-20

**Authors:** Anke Versluis, Kristell M Penfornis, Sven A van der Burg, Bouke L Scheltinga, Milon H M van Vliet, Nele Albers, Eline Meijer

**Affiliations:** 1 Department of Public Health and Primary Care Leiden University Medical Center Leiden Netherlands; 2 Unit Health, Medical, and Neuropsychology Institute of Psychology Leiden University Leiden Netherlands; 3 Netherlands eScience Center Amsterdam Netherlands; 4 Department of Biomedical Signals and Systems University of Twente Enschede Netherlands; 5 Roessingh Research and Development Enschede Netherlands; 6 Department of Intelligent Systems Delft University of Technology Delft Netherlands

**Keywords:** smoking, physical activity, virtual coach, eHealth, development, collaboration, conversational agent, risk factor, cardiovascular disease, CVD, digital, smoking cessation, intervention

## Abstract

Health care is under pressure due to an aging population with an increasing prevalence of chronic diseases, including cardiovascular disease. Smoking and physical inactivity are 2 key preventable risk factors for cardiovascular disease. Yet, as with most health behaviors, they are difficult to change. In the interdisciplinary Perfect Fit project, scientists from different fields join forces to develop an evidence-based virtual coach (VC) that supports smokers in quitting smoking and increasing their physical activity. In this Viewpoint paper, intervention content, design, and implementation, as well as lessons learned, are presented to support other research groups working on similar projects. A total of 6 different approaches were used and combined to support the development of the Perfect Fit VC. The approaches used are (1) literature reviews, (2) empirical studies, (3) collaboration with end users, (4) content and technical development sprints, (5) interdisciplinary collaboration, and (6) iterative proof-of-concept implementation. The Perfect Fit intervention integrates evidence-based behavior change techniques with new techniques focused on identity change, big data science, sensor technology, and personalized real-time coaching. Intervention content of the virtual coaching matches the individual needs of the end users. Lessons learned include ways to optimally implement and tailor interactions with the VC (eg, clearly explain why the user is asked for input and tailor the timing and frequency of the intervention components). Concerning the development process, lessons learned include strategies for effective interdisciplinary collaboration and technical development (eg, finding a good balance between end users’ wishes and legal possibilities). The Perfect Fit development process was collaborative, iterative, and challenging at times. Our experiences and lessons learned can inspire and benefit others. Advanced, evidence-based digital interventions, such as Perfect Fit, can contribute to a healthy society while alleviating health care burden.

## The Problem

The leading cause of disease burden across the globe is cardiovascular disease (CVD) [1]. Over the years, CVD prevalence and the number of CVD deaths have increased; in 2019, there were 523 million cases of CVD and 18.6 million deaths due to CVD [1]. CVD mortality is decreasing in most European countries, yet there are still 3.9 million deaths yearly [2,3]. Important behavioral CVD risk factors include smoking, low physical activity, unhealthy diet, and alcohol use [2,4]. These risk factors are modifiable and can help decrease the number of CVD cases and deaths [2,5]. Individuals with a low socioeconomic position (SEP) often have a less favorable profile of risk factors [6], resulting in a higher disease burden and premature death. Cost-effective interventions targeting 1 or more risk factors need to be implemented [1].

Interventions have been successful in initiating healthy behaviors (eg, physical activity) and stopping unhealthy behaviors (eg, smoking) [7,8]. However, these interventions have been relatively unsuccessful in attaining enduring healthy behavior [7,8]. For example, an intervention in individuals at risk for CVD had a significant positive effect on smoking cessation; however, this effect was not maintained at 1-year follow-up [9]. Behavior change is difficult for several reasons, including the fact that coaching or advice is not always available when the individual needs it most; in daily life, when encountering situations that may trigger a relapse. Even when an individual has frequent appointments with a health care professional, the individual is the only one who can be responsible for managing the behavior 24 hours a day and 7 days a week [10].

eHealth apps are increasingly used to support self-management [11,12]. One of the advantages of such apps is that they offer support whenever and wherever. Potential other benefits include accessibility, scalability, cost-effectiveness, and increased disease self-management [11,13]. However, standard or one-size-fits-all approaches appear less effective than personalized interventions [14,15]. This is understandable as each individual likely has different needs or wishes and might have a different preferred coaching style (eg, more or less directive). Therefore, when developing eHealth apps, it is important to identify how the app can be tailored to the needs of the individual users. Not only are eHealth apps often static, but they are also frequently developed without (sufficiently) engaging end users [16,17]. This is especially problematic as many existing interventions do not sufficiently meet the needs of low SEP individuals [18-20] and, consequently, can increase health inequalities. Involving end users, including those from lower SEP groups, and other relevant stakeholders can help optimize the adoption and adherence to the eHealth intervention [16,21] and result in the maintenance of healthy behavior.

## The Solution: Advanced Digital Support for Behavior Change

This paper describes the development of the Perfect Fit intervention. This eHealth app targets 2 key CVD risk factors; that is, it aims to support individuals at risk of CVD to stop smoking and increase their physical activity using a virtual coach (VC). Smoking and low physical activity are targeted because both are still highly prevalent in Europe. Importantly, the 19% smoking prevalence and the fact that the majority of Dutch adults are not sufficiently physically active, show that existing approaches do not reach their goals [22,23]. Considering that smoking cessation and increasing physical activity can reduce the risk of CVD and other chronic diseases, the 2 behaviors can be considered important targets for behavior change interventions [24-26]. Furthermore, smoking and insufficient physical activity often co-occur, especially among socioeconomically disadvantaged and ethnic minority groups [27], thereby increasing both the incidence of CVD in these groups and health-related inequities.

Interventions that target multiple health risk behaviors are both promising and present challenges. Among these challenges lie decisions regarding whether to promote health-promoting behaviors (like physical activity) or discourage health-compromising ones (like smoking), and whether to intervene in these behaviors sequentially or simultaneously [28]. In Perfect Fit, the decision was made to encourage physical activity and smoking cessation simultaneously. Reasons for this include that, on one hand, smoking cessation can help make physical activity easier, as ex-smokers’ physical fitness can improve quickly [29]. On the other hand, physical activity promotion might aid smoking cessation, potentially because physical activity can help reduce cravings and thereby prevent relapses [30-32]. Furthermore, simultaneously targeting multiple behavioral risk factors can help reduce the global disease burden [5]. By targeting both behaviors at once, we believe that synergy can be created in preventing CVD. To reach those most in need, the Perfect Fit intervention is specifically targeting individuals with a low SEP.

Perfect Fit builds on evidence that SMS text message–based interventions and conversational agents (like a VC) are promising for smoking cessation [33-35]. In addition, Perfect Fit incorporates evidence-based behavior change techniques as well as novel strategies to facilitate identity change (eg, toward becoming a nonsmoker or physically active person) [36-39], sensor data to objectively assess physical activity and set a personalized activity goal [40], and tailoring of motivational strategies to the user [15,41].

The main theoretical frameworks underlying Perfect Fit are the self-regulation theory [42-45], the PRIME (plans, responses, impulses, motives, and evaluations) theory [36], and the relapse prevention model [46,47]. Self-regulation theory considers all behavior goal-oriented, and successful behavior change occurs only when it is meaningfully linked to higher-order goals that represent personal values or self-concept elements [48]. Central to the relapse prevention model are high-risk situations; that is, situations associated with unhealthy behavior, such as negative emotional states or social pressure. Planning how to avoid such situations and how to adaptively cope with them if they occur is essential. Actual experience with adequate coping increases mastery of feelings, which in turn reduces the chances of lapsing. Nevertheless, lapses are not considered failures, but learning experiences that provide insight into unique individual challenges that need to be dealt with in future situations. Lapses thus represent only temporary threats to a sense of control. Identity theories such as PRIME theory [36] expand on the role of identity in behavior, stating that people are motivated to behave in line with how they perceive themselves, such that identity is a stable guide for behavior. Empirical work has clearly shown that people need to be able to see themselves as nonsmokers, and less as smokers, to quit smoking successfully [39,49-54]. An in-person identity-based intervention, which simultaneously targeted physical inactivity and smoking showed significant increases in runner identity and behavior and decreases in smoker identity and behavior [55,56]. It was chosen to monitor steps and set adaptive step goals as personalized goals help more in increasing physical activity compared with static goals (ie, 10,000 steps) [40,57]. Adaptive goals may not lead to immediate changes like static goals but they promote a more gradual and likely more sustainable increase in physical activity. Furthermore, personalized goals are perceived as more achievable by users, making them more motivating and engaging.

Tailoring of motivational strategies is not only based on more or less fixed user characteristics (eg, age, household size, and personality) but also the states users are in (eg, motivation, self-efficacy, and knowledge). While user characteristics such as personality [58], need for cognition [59], and cultural background may influence the effectiveness of motivational strategies [60], user states may also play a role. For example, Bertolotti et al [61] showed that self-efficacy can affect the effectiveness of messages encouraging healthy eating. Considering user states may be especially helpful when it comes to optimizing users’ reactions to motivational attempts in the long run. This is the case because motivational attempts can, in turn, also affect users’ states and thus the effectiveness of future motivational attempts. For instance, differently framed smoking cessation messages can differ in their effect on self-efficacy [59]. Thus, by considering not only current but also future user states, we might be able to choose motivational attempts that are more effective in the long run. Perfect Fit, therefore, also adapts motivational strategies to current and future states. In doing so, we build on previous work on adapting to current and future user states in context such as choosing the timing of tooth brushing reminders [62] and suggesting step goals [63].

## Approach

We will describe the development of the Perfect Fit intervention by giving an overview of the different research activities and methods used by the interdisciplinary research team. Altogether, these activities helped us identify how an eHealth intervention can best support smoking cessation and physical activity promotion in individuals in need, especially in people with a low SEP. In addition, the outline of the intervention journey will be presented, as well as lessons learned from the development process. These lessons learned can inform and support researchers and other stakeholders in collaborating effectively within an interdisciplinary team and on how to develop an (eHealth) intervention for a low-SEP population using different methods. Although this has been described for other types of interventions [64,65], we are unaware of similar developmental intervention studies for digital interventions addressing smoking cessation and physical inactivity combined.

### Design

Perfect Fit is a 5-year project in which academics from various research fields collaborate with public and private partners, end users, and health care professionals. This paper reports on 6 approaches used in parallel during the project’s first 2 years to develop an optimal eHealth intervention to support smoking cessation and physical activity.

### Six Integrated Approaches

**Figure 1:**
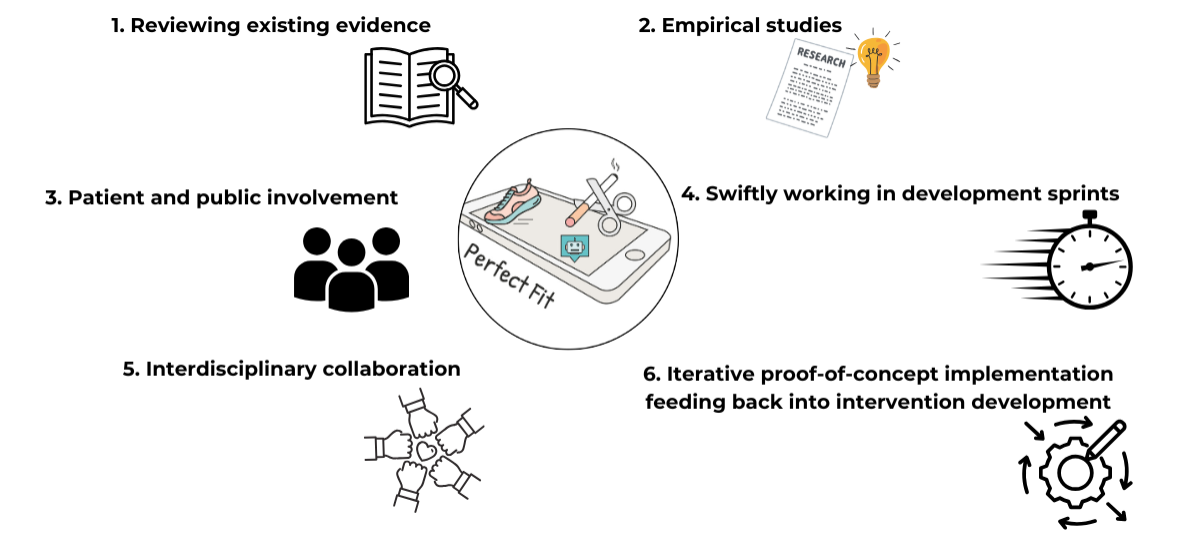
The 6 integrated approaches used to develop the eHealth intervention Perfect Fit.

The 6 approaches are shown in Figure 1. Below, we outline what was done in each of the 6 approaches.

#### Approach 1: Reviewing Existing Evidence

Scientific and gray literature on existing evidence in relevant research domains (eg, health and behavioral psychology, data science, and biomedical and computer engineering) is continuously reviewed within the Perfect Fit research group. Scientific evidence is reviewed at the start of every new empirical study, which allows us to identify what is known about a specific research topic, identify possible gaps in current knowledge, and put study findings into perspective.

##### Rapid Reviews of Smoking Cessation and Physical Activity Promotion

At the start of the Perfect Fit project, multiple rapid reviews were conducted. In total, 2 such reviews looked at existing commercial and empirically-tested smoking cessation apps (eg, SmokeFree [66] and Stopcoach [67]) and physical activity enhancing apps (eg, Accupedo-Pro Pedometer [68] and Samsung Health, developed by Samsung Electronics [69]). For each app, features and behavioral change strategies were identified, and strengths (eg, free of charge) and limitations (eg, steps are only counted when carrying your phone) were based on user reviews and, when available, empirical findings. In addition, 4 other rapid reviews looked at (1) existing wearables and their use in practice to track physical activity and breathing movements, (2) which measures and associated measurement tools are used in the literature when it comes to physical activity and smoking, (3) what measures exist to determine user engagement, and (4) how high-risk situations for smoking and physical inactivity can best be identified and acted on. All reviews were discussed within the Perfect Fit research group to determine which components to integrate into the Perfect Fit intervention.

##### Systematic Reviews of Scientific Literature to Ensure an Evidence-Based Intervention

A rapid review of systematic reviews and meta-analyses allowed us to identify the behavioral change techniques [70] most commonly used and reported as most effective in interventions that promote smoking cessation and physical activity. In addition, multiple systematic literature reviews are currently in progress to gain an in-depth understanding of specific research topics relevant to Perfect Fit [71]. To illustrate, the Perfect Fit intervention includes intervention components based on identity theories (eg, studies by West and Brown [36] and Kearney and O’Sullivan [72]), which posit that identity (ie, how one sees themself) is a determinant of behavior. To ensure the empirical soundness of the intervention components related to identity, a systematic scoping review and a systematic review are being conducted to understand the role that identity plays in both behaviors and to identify the mechanisms through which identity motivates smoking and physical activity behavior (change) in existing interventions. Literature reviews conducted so far have provided valuable (evidence-based) information concerning important components, coaching techniques, mechanisms, theories, attributes, and functionalities to consider and include in the Perfect Fit app.

#### Approach 2: Empirical Studies

We conducted empirical studies to test and gather input for (1) intervention components, (2) the VC, and (3) the personalization of intervention components and VC. For example, for input on the intervention components, we have experimentally examined the effect of future-self exercises on smoking-related self-identity constructs [49], and explored the experience of insufficiently active individuals with a low-to-middle SEP with future-self exercises. Furthermore, we have validated a long-term goal-setting dialog with a VC for running or walking based on its effect on self-efficacy [73], preparatory activities for quitting smoking and becoming more physically active based on people’s experiences with and effort spent on them [41,74], and motivational messages based on how motivating they are perceived in the context of physical activity [75]. All these studies were conducted online, facilitating reaching a large and diverse audience in their natural environment. We are also running a virtual reality study, in which daily smokers with an intention to quit smoking are shown an environment that is associated with smoking. We compare 3 smoking lapse prevention dialogs with a chatbot on their effects on abstinence self-efficacy, phasic craving, and affect and examine the overall user experience and acceptability of the chatbot.

Since users of the Perfect Fit intervention will ultimately be guided by a VC, we have also studied specific elements of such a VC. The focus has been on people’s attitudes toward preliminary versions of a VC they interacted with, as interacting with an app helps identify barriers and benefits to using it [76]. These preliminary versions of the VC incorporated ideas from motivational interviewing [77], focused on maintaining a positive and encouraging attitude, reduced repetitiveness of the utterances [78], and aimed to make conversations accessible for people with low literacy levels by breaking up large chunks of text into multiple messages and letting people indicate when to continue. To get input on multiple design ideas, we have also examined people’s views on videos of interaction scenarios for a VC [74]. For example, people were asked whether they would want to reflect on difficult situations concerning quitting smoking that occurred during the day with their VC in the evenings. Importantly, participants had previously interacted with a preliminary VC for about 2 weeks to account for the novelty effect [79,80].

A core feature of our intervention is that it is personalized to match the needs of individual users. Several of our empirical studies have investigated ways of personalizing the intervention as a whole and the coaching by the VC. For the intervention, we have conducted preliminary studies on how sensors can be used for personalization After an evaluation of algorithms for offering a personalized step goal, the most suitable algorithm was implemented [57]. In addition, this algorithm was tested and evaluated on 117 participants who collaboratively set daily step goals with their text-based VC Steph for up to 5 consecutive days [81]. As the Perfect Fit intervention makes use of sensor data, its effects on cardiorespiratory fitness can be estimated using collected step count and heart rate data [82]. Regarding personalized coaching, we have taken the first steps to examine how to make goal setting, data monitoring, and the assignment of preparatory activities personalized and adaptive [83]. We have already tested approaches using techniques such as linear regression models [73], rules derived from behavior change theories and experts [75], and reinforcement learning [41,81]. While our initial results are promising, we also gathered data to improve the approaches. For example, in a longitudinal study to assess the effectiveness of a reinforcement learning algorithm for persuading people to do preparatory activities for quitting smoking, we collected data on more than 30 user characteristics (eg, gender, physical activity identity, and need for cognition) [84] that can be used to tailor the intervention to (groups of) users. While it seems that accounting for such user characteristics that can be measured before an intervention is less effective than accounting for users’ current situations or states (eg, motivation, presence of reminders, and knowledge), there might be a benefit of considering user characteristics in addition to states [85]. In an interview study with experts who coach individuals to quit smoking or increase their physical activity, we have further learned how and to what extent they adapt their coaching techniques to the individual that they coach.

#### Approach 3: Patient and Public Involvement

The results of the empirical studies conducted in a large and diverse audience (approach 2) contribute to the development of an intervention that can be used by a broad audience. Still, one of the aims of the Perfect Fit project is to make the intervention also accessible and relevant for individuals with a low SEP. Therefore, this specific population is involved in different ways throughout the project. Their involvement is essential because those using the intervention can best advise on the research’s relevance and the intervention’s usability [86].

An advisory panel with potential end users of the Perfect Fit app was set up 1 year after the start of the project. This panel consists of 4 members who have experience with quitting smoking or the intention to quit smoking and want to be more physically active. The panel will be involved during the whole project and is consulted in several stages of the project and for several substudies. This way, the advisory panel can represent the perspective of the target population, and the rest of the research team can ensure that this perspective is integrated into research materials and the final intervention. Enriching a research project team with an advisory panel can help empathize with the target population’s perception and match study procedures, materials, and the developed intervention to their needs, wishes, and skills. This will increase the likelihood of successful adoption and the effectiveness of the Perfect Fit intervention [86,87].

Next to the recurring involvement of the advisory panel, other one-time patient and public involvement activities were organized within the Perfect Fit project to receive more extensive input on specific intervention components. For instance, 3 focus groups were held with physically inactive older individuals (aged 45 years and older) with a low-to-middle SEP to discuss their experience with and opinion on possible components of the Perfect Fit intervention.

Other stakeholders outside of the direct target audience of the Perfect Fit intervention were also consulted. For example, interviews have been conducted with experts (ie, lifestyle coaches, psychologists, physical therapists, smoking cessation coaches, and practice nurses) to ensure that their expertise in coaching individuals to quit smoking and increase their physical activity would also be included in the intervention.

#### Approach 4: Swiftly Drafting Dialogs in Content Development Sprints

Designing dialogs between a user and a VC is challenging. First, it is inherently interdisciplinary. On one hand, psychological expertise and affinity with the target group are needed. On the other hand, technical expertise is desired. Specifically, human-machine interaction competence and insight into technical feasibility are vital. Second, bridging the gap between an abstract list of requirements and developing actual content that fulfills these requirements is a challenging creative process.

To accommodate these challenges, we organize regular content development sprints. First, the content team develops dialogs, which is directly followed by a software development sprint of about 2 weeks led by the technical team. The 2 teams continuously consult one another if necessary to allow for a rapid development and implementation, facilitated by daily stand-up meetings during the software development sprints. At the end of the sprint, we demonstrate the new content to key stakeholders for feedback. These sprints are inspired by Scrum [88].

Sprints took place after the intervention was roughly sketched out and during the development of the first prototype of the system. More sprints will be organized to finetune the content during the development of the final product. By working together in sprints, design decisions can be made swiftly. It can help to ensure that the created content meets the functional requirements and is technically feasible. Importantly, it is useful to implement and test the dialogs in a prototype app as soon as possible, and to involve end users in this process. This way, lessons learned from testing the first dialogs can be used in the upcoming content development process.

#### Approach 5: Interdisciplinary Collaboration

Working with multiple disciplines is vital when designing and developing a VC for health behavior change. In the Perfect Fit project, the disciplines that work together are health and clinical psychology, human-computer interaction, biomedical engineering, data analytics, and software engineering. Because of the high interdisciplinary degree of this research project, there are a lot of different experts on specific topics within the project. Therefore, frequent and clear communication between disciplines is crucial. The core of this communication plan is monthly meetings with all academic partners to discuss all project developments and weekly meetings with the technical and content teams. These frequent meetings ensure that the ideas developed by the psychological team match the technical feasibility and that technical developments align with the requirements from the psychological perspective. Several documents and tools were used for input of the various meetings. For example, a MoSCoW (must have, should have, could have, and will not have) prioritization [89] was used where all partners filled out their requirements and the priority of these requirements (refer to Multimedia Appendix 1). Furthermore, an intervention journey was created and extensively discussed. This intervention journey consists of a timeline with all the important events a user would experience (refer to Multimedia Appendix 2). It includes both in-app and out-of-app events and is the intervention blueprint. In addition, content development sprints are performed; a short period with intensive collaboration between a technical and psychological expert to develop a dialog (refer to Approach 6 section for more details). All content included in the Perfect Fit intervention has been developed through an iterative feedback process by numerous stakeholders within (eg, academics from various backgrounds) and outside (eg, professionals) the Perfect Fit research team. Collaboration between disciplines also facilitated the empirical studies (refer to Approach 2 section); for example, the technical team could use the psychological expertise of others to improve the development of algorithms. Furthermore, a virtual reality system was set up by the human-computer interaction team and could be used by psychology experts to test newly developed dialogs with end users [90].

In addition to academic partners, The Perfect Fit consortium has public and commercial partners with valuable knowledge. Multiple expert sessions with these partners were organized. For example, the national expertise center Pharos was consulted for their expertise in health disparities so that the developed intervention would reach and fit the needs of the low-SEP group (ie, the intervention will be available for everyone, but we wanted to make sure the app was specifically usable for the low-SEP group). SenseHealth is experienced in developing health care and chat apps, and their technical expertise was used for the software architecture. Roessingh Research and Development specializes in monitoring physical activities using ambulatory sensors and their expertise is used in coupling sensors to collect data and their equipment is used for data collection for algorithm development. These meetings, as well as the annual consortium meetings, contribute to a better overview of the user requirements and technical possibilities. In addition, the output of the meetings feeds back into the content development sprints (refer to Approach 3 section).

#### Approach 6: Iterative Proof-of-Concept Implementation That Feeds Back Into Intervention Development

We implemented a working proof-of-concept technical implementation of the intervention as soon as possible after the initial design phase. This was done in sprints similar to the content development. The technical team committed to delivering a certain number of new features in 2 weeks, working together intensively. After each sprint, these new features were presented to the rest of the Perfect Fit team. In addition, the intermediate products were tested by the advisory panel (refer to Approach 3 section). Feedback from the Perfect Fit team and advisory panel often led to drastic design changes and improved consensus on the intervention requirements. For example, certain dialogs were changed to videos, because they were considered more user-friendly and, moreover, the technical implementation of videos fitted better in the planning of the technical team. During this process, we also involved privacy and security officers to ensure that the application complies with all applicable laws, such as the General Data Protection Regulation. In our experience, having a concrete proof-of-concept implementation is critical when working toward an efficient real-world implementation of the intervention. This process was inspired by Agile software development [91].

## Lessons Learned: Development Process and Intervention

The lessons learned from the project are presented in Table 1 and Table 2. Table 1 shows the lessons learned from the development process (eg, collaboration and technical development), and Table 2 shows the lessons learned from the Perfect Fit intervention. For each lesson learned, we added the specific approach or approaches that contributed to the lessons learned. The app requirements using MoSCoW framework, intervention journey, and a visualization of the “high-risk situation” dialog are presented in, respectively, Multimedia Appendix 1, Multimedia Appendix 2, and Multimedia Appendix 3.

The intervention was developed to be tested in a proof-of-concept trial [92,93]. The open-source software is available through GitHub [93], and the dialogs and exercises used in the intervention are available digitally [94]. Technical documentation, including a diagram of the software architecture, can be found on the Internet [95].

**Table 1:** Overview of the lessons learned from the Perfect Fit development process.

Lesson learned and the approach or approaches that contributed to this		Explanation
**Collaboration with stakeholders**		
	Involve all relevant interdisciplinary expertise and use iterative feedback—5 and 6	To ensure the content is appropriate, involve research members with relevant expertise from multiple disciplines, and use an iterative feedback process when writing, reviewing, and improving the content.
	Facilitate multidisciplinary collaboration—4, 5, and 6	To facilitate collaboration, schedule regular meetings, and select a communication platform to share knowledge and work on projects.
	Make clear agreements with the stakeholders—3 and 5	Make agreements on the roles of stakeholders (eg, research team and advisory panel members), manage expectations, evaluate, and be transparent about what is done with feedback from the stakeholders.
	Involve the target population early on—3	Plan the involvement of the target population early in the project so that you can account for it in the planning and budget. This also ensures that the perspective of the target population is taken into account in an early stage (eg, when formulating research questions) and that the involved individuals get to know the project well.
	Ensure that all members of the advisory panel are trained—3	Ensure that members of an advisory panel feel capable of sharing their opinion and that the research team can collaborate with them. Provide training for advisory panel members and researchers if necessary. Also, make the advisory panel feel invited to share their opinion.
**Technical development**		
	Intervention development requires a continuous feedback cycle—4 and 5	Testing and demoing a minimal but working prototype as soon as possible is vital. Feedback from stakeholders should be used continuously to update the design. Development requires substantial time and effort, which is preferably budgeted.
**Other**		
	Understand to better implement—1 and 5	Make critical summaries of available evidence to understand relevant theories and mechanisms, as this facilitates the development and implementation of intervention components.
	Literature reviews facilitate decision-making—1 and 5	Summarizing successes, lessons learned, and limitations of existing apps and interventions can facilitate decision-making in a research group (eg, which components to integrate into an intervention).

**Table 2:** Overview of the lessons learned from the Perfect Fit intervention.

Lesson learned and the approach or approaches that contributed to this			Explanation
**Tailoring**			
	Tailor the timing and frequency of intervention components—2 and 3	Preference for the timing and frequency of the intervention may vary, which requires tailoring to the individual user [74]. Reminders may help those who forget to do or complete an activity [74].	
	Some activities are not suitable for everyone—2 and 3	Not all intervention components are suitable for all [74,96]. For example, a visualization exercise can be unsuitable for those who struggle with mental imagery. It is therefore vital to include various exercises and to provide flexibility regarding which one should be done by users. Or allow people to modify activities, such as by visualizing a soccer match or bike race instead of a fighting match [74].	
	People may be familiar with certain activities—2	People may have done specific activities before [96], which can affect activities’ perceived difficulty and usefulness. Ideally, this should be taken into account when suggesting activities or allowing users to choose between activities.	
	Tailor motivational messages—1 and 2	Motivational messages tailored to mood, self-efficacy, and progress are more motivating than general messages [75]. Reproducible tailored motivational messages for the VC^a^ can be generated by asking experts to write messages for scenarios with a structure derived from an ontology [75]. Furthermore, considering the user and their current state (eg, available time and self-efficacy) is helpful when choosing a persuasive strategy [41].	
**VC**			
	Respect the autonomy of individuals—1, 2, and 3	People’s autonomy can be violated when a VC recommends help to users [74]. For example, people may oppose a recommendation formulated like a command instead of a suggestion. Therefore, consider not only what is recommended, but also when and how. Test the appropriateness of recommendations with end users.	
	Think carefully about how the VC responds to people’s answers—1 and 2	Perceiving the VC as caring and empathetic can make people more satisfied with the VC. Repetitiveness of dialogs can harm this perception. Also, default answers (eg, thanks for letting me know) can be inappropriate when users talk about serious experiences. It may harm the relationship and can be considered frustrating. Furthermore, closed-ended questions should have all possible answer options to ensure a fitting response can be chosen [97]. The language should also be appropriate (eg, not too enthusiastic).	
	Allow users to correct their answer—2	People should be able to correct their answers. When not given this option, people may write about earlier entry errors when responding to later (free text) questions [96].	
	Positive attitudes toward VC—2	A VC design based on motivational interviewing techniques, with a positive and encouraging attitude, limited repetition, multiple short messages instead of large texts, and letting people indicate when to continue can lead to positive attitudes toward the VC [73,97].	
	Let users “get to know” the VC—1 and 2	Some participants may want to “get to know” the VC [97]. It is thus recommended that the VC discloses something about itself, while ensuring that the user knows they are interacting with a VC and not a human [98].	
**Intervention components**			
	Need for identity-based exercises in smoking cessation interventions—1 and 2	Identity and especially nonsmoker identity are important determinants of smoking behavior and should be included in smoking cessation interventions [49]. A study also showed that identity-based exercises were motivating [74].	
	Limited effect of the future self exercises in their current form—2	In their current form, the future-selves exercises were not successful in changing identity and behavior. These exercises need to be improved (eg, repeated or made longer) [49].	
	A goal-setting dialog for physical activity with a VC that provides examples of other people in its current form decreases people’s self-efficacy, but may lead to more realistic evaluations of abilities—2	A goal-setting dialog that uses (personalized or generic) examples of other people who increased their physical activity decreases self-efficacy [73]. However, this could help people to realistically evaluate their abilities. It is important to strengthen self-efficacy when setting goals.	
	Importance of perceived usefulness of intervention components—2	Perceived usefulness was the most important theme in people’s free-text responses about experiences with preparatory activities and views on interaction scenarios for a VC [74]. The perceived usefulness of an intervention component may also differ between people [74]. Intervention components should be designed with their perceived usefulness in mind, with a potential need to tailor to (groups of) users.	
**Other**			
	Keep it simple, appropriate for all, and user-friendly—1, 3, and 5	Do not use too much text. Use images, infographics, and short videos instead. Avoid making statements or assumptions that may not apply to everyone and phrase carefully (“perhaps you notice” instead of “you will notice”). Maximize user-friendliness and efficiency of the intervention by, for instance, minimizing the number of steps users have to go through, having easy-to-use functions, and sending notifications on when to take action.	
	Make your reasoning and expectations clear—3 and 5	Explain what you expect from the user and why. It can increase user engagement.	
	Provide clear explanations of activities and give feedback—2	People may struggle to notice the differences when activities are similar (but not the same) [96]. Clearly explain the activity and how it is different from other activities. Provide feedback on completed activities, as users want to know whether they have done it correctly [74,96].	
	Explain the preparation phase and tailor its length—2	In our study [74], several participants quit smoking or became more physically active or both even though they were just asked to prepare for these changes. This suggests that the preparation phase needs to be clearly explained. Also, the length of the preparation phase should be tailored to the user.	
	Align end-user preferences with applicable regulations—3 and 6	Preferences of users may conflict with security or privacy regulations. In our case, users preferred a widely available commercial messaging service as a front-end, which would lead to privacy and security problems. When such a conflict arises, identify appropriate alternatives that are acceptable to all.	

^a^VC: virtual coach.

## Reflections and Future Directions

It is well-known that smoking and insufficient physical activity put people at risk of CVD and other adverse health outcomes [2,4]. However, changing and maintaining healthy behavior is challenging, even if people know why this is important and are motivated to do so [8]. It is particularly challenging for people with a low SEP, because stressful life circumstances may complicate behavior change processes. In addition, many existing interventions do not sufficiently meet their needs [18,20]. Effective interventions are needed to help people reach and maintain a smoke-free, physically active life. Importantly, these interventions must align with the needs of those who need intervention the most [17]. Given that smoking and physical inactivity are highly prevalent and the health care system is under pressure in many countries, interventions with a broad reach that support individuals wherever, whenever, and however an individual needs it are desired. For this reason, the Perfect Fit consortium undertakes a 5-year endeavor to develop and test an innovative eHealth intervention for quitting smoking and increasing physical activity, targeted at people at risk of CVD and designed to meet the needs of those with a low SEP in order to reach a broad audience.

This article describes the Perfect Fit intervention’s development process and lessons learned from the development process and the Perfect Fit intervention. We first identified what was already available in the fields of health psychology, human-computer interaction, data science, and biomedical engineering. We further expanded the already available knowledge with empirical studies in these respective fields to gain insight into what was unknown and test our ideas early on.

The resulting knowledge was used to develop intervention content, for example, when drafting VC dialogs in content development sprints. We quickly implemented new ideas and content in the emerging software and technical architecture such that the developing intervention could be evaluated and improved through an iterative approach. At the same time, we collaborated with end users with a low SEP to ensure that this sophisticated intervention was accessible, understandable, and easy to use. This process was highly collaborative, iterative, and at times complicated. We hope to inspire others and provide guidance throughout this challenging process by presenting the intervention we developed and the lessons learned.

The next step for the Perfect Fit intervention is conducting a proof-of-concept study. When the results are positive, preparations for making the intervention available beyond this study will be made. Specifically, a mixed-method study with a pre-post test design will be used to assess the intervention’s acceptability, usability, and preliminary effectiveness in helping people quit smoking and increase their physical activity. The intervention will target smokers at risk of developing CVD, and we aim to include as many low SEP smokers as possible. The duration of the intervention is personalized, but the average duration is expected to be 16 weeks. Study outcomes will be assessed at baseline, postintervention, and at 3 follow-up moments (ie, 2, 6, and 12 months after postintervention). The study is registered on ClinicalTrials website (NCT06095999). This study will increase our understanding of personalized VC-based interventions, and of multi-health behavior change interventions that simultaneously target a health-promoting and a health-compromising behavior. In parallel to the proof-of-concept study, we are conducting a small-scale feasibility study in 2 mental health care institutes to examine the technical and commercial feasibility and societal impact of sustainably implementing Perfect Fit, including the requirements to do so. Second, a stakeholder analysis with relevant public-private partners will be done to develop a clear business plan. This can help with the sustainable implementation of the innovation and prevent the innovation from ending in the “valley of death” (ie, the metaphorical place where many technologies end up after research funding is finished) [99,100], and instead can make a difference by helping people live healthily.

Besides testing the Perfect Fit intervention and preparing for its implementation, we are also further developing and testing the intervention components, the VC, and their personalization in separate empirical studies. For example, recognizing the importance of taking users’ usefulness beliefs into account when proposing activities to them [74], we are developing an algorithm for proposing preparatory activities for quitting smoking that accounts for the perspectives of both users and experts [101]. Furthermore, while users are currently simply given a daily step goal by their VC, users should ideally have a say in setting these daily step goals. We thus designed a daily goal-setting dialog in which users collaboratively set daily step goals with their VC [81]. The initial step goal proposal the VC makes is thereby based on a recommended goal that is derived from the previous activity of the user and adjusted based on a reinforcement learning algorithm that accounts for users’ current and future states (eg, mood and motivation). Our findings from human data–based simulations show that the initial step goal proposal matters and that choosing an optimal one based on the reinforcement learning algorithm could increase the probability that users move to a favorable next state in which they are more likely to achieve their previous activity-based recommended step goal. Third, we go beyond the idea of an intervention that relies on purely virtual coaching by investigating how we could add human coaching to make people feel more accountable to the intervention [74,102,103]. Specifically, we are designing a reinforcement learning algorithm that considers current and future user states to determine when a human coach should best give feedback to users. Previous work by Piette et al in the context of pain management [104] and reducing opioid-related risks [105] has shown the promise of considering user states in determining when to involve a human coach. However, we go a step further by not only considering resource constraints (eg, a human coach is available for at most 2 h per day) but also accounting for different ethical principles for allocating sparse medical resources [106]. Taking a blended approach might not only improve the commonly low adherence rates to eHealth apps for behavior change [107], but also make other stakeholders see the intervention more favorably, thus further increasing the chance of sustainable implementation of future versions of the Perfect Fit intervention.

## Appendix

Multimedia Appendix 1 Application requirements for Perfect Fit.

Multimedia Appendix 2 Overview of the Perfect Fit intervention.

Multimedia Appendix 3 Visualization of the “high-risk situation” dialog.

## Abbreviations

CVD: cardiovascular disease
MoSCoW: must have, should have, could have, and will not have.
PRIME: plans, responses, impulses, motives and evaluations
SEP: socioeconomic position
VC: virtual coach
