# Supplementary material for: Targeting Key Risk Factors for Cardiovascular Disease in At-Risk Individuals: Developing a Digital, Personalized, and Real-Time Intervention to Facilitate Smoking Cessation and Physical Activity
Source: JMIR Cardio. 2024 Dec 20;8:e47730. doi: 10.2196/47730 (PMC11699499; doi:10.2196/47730)
Supplement: Multimedia Appendix 1 [file cardio_v8i1e47730_app1.pdf]

Multimedia Appendix 1

Application Requirements Perfect Fit

To get an overview of and prioritize application requirements, we proceeded in the following steps:

1. Each research partner wrote down requirements they had.
2. Requirements were divided into 15 categories to get a better overview (e.g., *Communication*, *User Profile*). Requirements can also appear in multiple categories, in which case we note this in the “Other Categories”-column.
3. Each main research partner (University of Twente, Leiden University/Leiden University Medical Center, Delft University of Technology, and the Netherlands eScience Center) assigned priorities to the requirements using the MoSCoW framework (must have, should have, could have, won’t have).
4. The final priority for a requirement was determined as the highest priority assigned by any research partner (green for “must have”, yellow for “should have” and orange for “could have”).
5. Dependencies between requirements were noted. This is especially helpful to ensure that all dependencies of “must have”-requirements are also assigned with the priority “must have.”
6. The technical team assigned levels of implementation difficulty to each requirement. The levels are 1) Already existing, 2) Implemented soon, 3) Relatively easy, 4) Much work, and 5) Not possible. Sometimes it was also not yet clear what the difficulty level was, in which case we entered a question mark. Levels 1 and 2 exist because our application is built upon the existing NiceDay application. So requirements that already exist are ones that are already implemented in NiceDay.

Below we show the requirements for two categories (*Communication* and *High Risk Situation*), together with their priorities assigned by the research partners, implementation difficulty levels, and dependencies. Notably, the requirements, their priorities, and their implementation difficulty levels are not fixed. Instead, they are updated throughout the project to reflect new insights (e.g., time requirements).

1. Communication

#	Requirement	Other Categories	Priority (Twente, Leiden, Delft, eScience)	Implementation Difficulty Level	Dependencies

1.1	Real-time text-message communication between virtual coach and human user	-	M, M, M, -	1	1.2
1.2	Virtual coach can send and receive messages (bi-directional)	-	M, M, M, -	1	
1.3	Possibility to ask the user closed-ended questions within system (e.g., cigarette consumption, physical activity, feelings, "who are you with when you have a craving?")	-	M, M, M, -	1 (in conversations with virtual coach)	
1.4	Identity-card creation ("What labels do you associate with who you are?")	User profile *(See 3.3)*	C, M, -, -	2	
1.5	Virtual coach and end-user able to access uploaded materials (e.g., images, motivational quotes, videos)	Data storage *(See 4.8)*	C, S, C, -	1 (text), 2 (images)	4.7
1.6	Onboarding/intake during which virtual coach and coachee get to know each other	User profile *(See 3.6)*	S, S, -, -	3, 4	1.1, 1.2
1.7	Real-time voice message communication between virtual coach and human user	-	C, C, C, -	?	
1.8	Real-time video communication between virtual coach and human user	-	C, C, C, -	1	
1.9	Possibility to receive outside support through the application (e.g., buddy, family)	-	C, C, -, -	3, 4	
1.10	Evaluation message of last physical activity to user based on data (e.g., "Well done, this workout was beneficial for your heart")	-	C, -, -, -	3, 4	1.2

2. High Risk Situation (HRS)

#	Requirement	Other Categories	Priority (Twente, Leiden, Delft, eScience)	Implementation Difficulty Level	Dependencies
2.1	User-driven identification of high-risk situations (HRS) (bare minimum: end-user names HRS)	-	M, M, C, -	3, 4	
2.2	System-driven identification of smoking HRS (timeframe, mood, plans)	-	M, M, M, -	3, 4 (depends on gathered data)	1.3

2.3	System-driven identification of physical activity HRS (timeframe, mood, plans)	-	M, M, C, -	3, 4 (depends on gathered data)	1.3
2.4	User-driven "Help" / "Danger"-function when in an HRS with answer from virtual coach	-	S, M, M, -	1 (in conversations with virtual coach), 2 (button)	1.1, 1.2
2.5	Personalized answer from virtual coach in an HRS	Personalization (*See 6.5*)	C, S, S, -	3, 4	1.1, 1.2, 2.1, 2.2, 2.3
2.6	Personalized (pre-recorded) answer from virtual coach in an HRS	Personalization (*See 6.7*), Theoretical basis (*See 7.3*)	C, C, C, -	3, 4	1.1, 1.2
