# Supplementary material for: Targeting Key Risk Factors for Cardiovascular Disease in At-Risk Individuals: Developing a Digital, Personalized, and Real-Time Intervention to Facilitate Smoking Cessation and Physical Activity
Source: JMIR Cardio. 2024 Dec 20;8:e47730. doi: 10.2196/47730 (PMC11699499; doi:10.2196/47730)
Supplement: Multimedia Appendix 2 [file cardio_v8i1e47730_app2.pdf]

Multimedia Appendix 2

Figure 1, on the next page, provides an overview of the Perfect Fit intervention, including the dialogs and videos presented to the user in each phase of the intervention.

The first, second and fifth column show the number, priority level and description of each element of the intervention. The priority level (column 2) is based on the MOSCOW list (see Multimedia Appendix 1). All the elements shown here are labeled as C1 (highest priority level; must-haves) as they structure the intervention and are necessary to prepare, support and coach users for long-term health behavior changes. The intervention consists of videos that the user can watch (e.g. C1.7), an audio fragment that the user can listen to (i.e. C1.11) and chat dialogs between the coach and the user (e.g. C1.5; see column 5).

Column 3 and 4 are meant to facilitate collaboration and show which team member is the main responsible person for developing a certain element (column 4) and which team members can be consulted during development (column 3).

The columns on the right show the intervention from start till closing. The duration of the preparation phase can be adjusted to users' preference (from a minimum of 11 days to a maximum of 3 weeks). The duration of the execution phase is at least 12 weeks, but can be extended when users experience relapse(s). The closing consists of only one dialog and this dialog will start after the last day of the execution phase.

A red 'x' indicates from which day/week a certain element can be presented to the user. During the preparation phase, the element will only be presented on day/in week 'x', given that the previous element is completed.

Figure 2, shown after Figure 1, gives an overview of the (optional) activities that can be presented to the user during the Perfect Fit intervention.

This overview shows the different activities (behavior change techniques) that can be done by the user during the intervention (column 5), which are labeled as C2 and C3 (lowest priority level) (see column 2). Three of the activities are presented to the user on day/in week 'x'. All the other activities can be chosen by the user on day/in week 'o'. However, these activities are optional.

Column 4 shows which individuals prepared the activities. Not all individuals mentioned (through abbreviations) are co-authors on the paper. Only individuals who made a significant contribution to the project and manuscript, were included as co-author.

Figure 1. Overview of the Perfect Fit intervention, including the dialogs and videos presented to the user in each phase of the intervention.

Perfect Fit (PF) - activity intervention planning

Number:	Priority level:	Consult team member:	Who takes care of this?	Description:	PREPARATION PHASE Week 1, Day ...							Week 2, Day ...							Week ...	EXECUTION PHASE Week ...												HRS & (RE)LAPSE	CLOSING Week 13, Day ...
					1	2	3	4	5	6	7	8	9	10	11	12	13	14	3	1	2	3	4	5	6	7	8	9	10	11	12		1
**MUST HAVE INTERVENTION COMPONENTS**																																	
PREPARATION PHASE																																	
C1.1	C1	-	MV	**Outside of app:** Introduction video about PF program, app and sensors																													
C1.2	C1	Technical team	MV	**Outside of app:** Informed consent																													
C1.3	C1	-	-	**Part of NiceDay already:** Register and create account in NiceDay	x																												
C1.4	C1	BS	KP	**Video:** Introduction preparation phase (e.g., meet your coach, congratulate on enrolling, explain PF program)	x																												
C1.5	C1	...	...	**Dialog:** Create profile (e.g., age, gender, preferences, settings, fill-in participant code)	x																												
C1.6	C1			**Video:** Medication talk (information about smoking cessation medication or aids)	x																												
C1.7	C1			**Video:** Track current behavior (e.g., gain insight into smoking behavior and physical activity, explain use of sensors)	x																												
C1.11	C1			**Audio:** Current and future-selves activity (e.g., visualize desired and feared future selves)	x								x								x						x						
C1.9	C1			**Dialog:** Goal setting (e.g., date for quitting smoking, physical activity goal, action planning)										x																			
C1.10	C1			**Video:** Fist-aid kit video (explain the PF first-aid kit, which can be consulted in difficult moments during program)										x																			
EXECUTION PHASE																																	
C1.15	C1			**Video:** Introduction preparation phase (e.g., congratulate for completing preparation phase, explain execution phase)																x													
C1.17	C1			**Dialog:** Weekly reflection (reflect on week, difficult moments, feedback goals, and prepare for next week)																x	x	x	x	x	x	x	x	x	x	x	x		
META DIALOGS																																	
C1.13	C1			**Dialog:** Rescheduling dialog 1 (short dialog to ask users if they want to complete upcoming dialog now or later)																													
C1.14	C1			**Dialog:** Rescheduling dialog 2 (short dialog to ask users if they want to complete upcoming dialog now or later)																													
C1.16	C1			**Dialog:** General activity dialog (reflect on previous activity and explain new activity; see 'optional activities')														x	x														
C1.12	C1			**Dialog:** Short dialog for presenting video's (e.g., to introduce and provide link to 'Medication talk video')																													
C1.8	C1			**Dialog:** First aid Kit (dialog/text which is shown when user consult their first-aid kit in a difficult moment)																													
(RE)LAPSE																																	
C1.19	C1			**Dialog:** User-initiated HRS or (re)lapse (provide help during difficult moment or (re)lapse, e.g., motivate user)																												x	
C1.18	C1			**Dialog:** System-initiated (re)lapse dialog for PA (tracked via number of steps; provide help, e.g., motivate user)																												x	
CLOSING																																	
C1.20	C1			**Dialog:** Closing (e.g., congratulate on achieving closing phase, reflect, make relapse prevention plan, say goodbye)																													x

Figure 2. Overview of the (optional) activities that can be presented to the user during the Perfect Fit intervention

Perfect Fit (PF) - activity intervention planning

Number:	Priority level:	Is there already some content available?	Who takes care of this?	Description:	PREPARATION PHASE Week 1, Day … 1	2	3	4	5	6	7	Week 2, Day … 8	9	10	11	12	13	14	Week … 3	EXECUTION PHASE Week … 1	2	3	4	5	6	7	8	9	10	11	12	HRS & (RE)LAPSE	CLOSING Week 13, Day … 1
**ACTIVITIES (BEHAVIOR CHANGE TECHNIQUES) WHICH CAN BE PERFORMED DURING INTERVENTION**																																	
Mind/Self-related activities																																	
C3.1	C3	Experiment KP	KP, PK	Herinnering aan je toekomstige zelf (Search for pictures/motto/quotes/a song which remind of envisioned future-self)																													
C2.1	C2	Presentation advisory board	KP, PK, FB	Reacties van vrienden en familie op je toekomstige ik (Envision how friends/family will receive your future-self)		o	o	o	o	o	o	o	o	o	o	o	o	o	o	o	o	o	o	o	o	o	o	o	o	o	o		
C3.2	C3	Experiment NA	KP, LC	Wedstrijdje in je hoofd (visualize smoking/physical activity as a battle and imagine yourself winning this battle)		o	o	o	o	o	o	o	o	o	o	o	o	o	o	o	o	o	o	o	o	o	o	o	o	o	o	o	
C2.2	C2	Stopcoach app	MV, PK, RE	Doe het anders (Think about alternative behaviors instead of smoking/doing a passive activity)														o	o	x	o	o	o	o	o	o	o	o	o	o	o	o	
C3.3	C3	Stopcoach app	MV, RE	Foto's van jezelf maken (Take a photo of yourself to see your appearance changing)		o	o	o	o	o	o								o	o			o			o			o				
C2.3	C2	Stopcoach app	KP, LC	Ontspanning (Think about relaxing activities to do, like mindfulness exercise, gardening, reading a book)		o	o	o	o	o	o	o	o	o	o	o	o	o	o	o	o	o	o	o	o	o	o	o	o	o	o	o	
C3.4	C3	…	…	Positieve zinnen (encourage to use positive self-talk)		o	o	o	o	o	o	o	o	o	o	o	o	o	o	o	o	o	o	o	o	o	o	o	o	o	o	o	
C2.4	C2			Terugdenken aan je succes (prompting focus on past quit success or other successes)		o	o	o	o	o	o	o	o	o	o	o	o	o	o	o	o	o	o	o	o	o	o	o	o	o	o	o	
C3.5	C3			Jij als voorbeeld (prompt identification as a role model, "What advice would you give others?")																			o	o	o	o	o	o	o	o	o		
C3.10	C3			Voet bij stuk houden (dealing with being the one who wants to change their behavior when others do not)		o	o	o	o	o	o	o	o	o	o	o	o	o	o	o	o	o	o	o	o	o	o	o	o	o	o		
Educational activities																																	
				Veranderingen in je lichaam (education about physical transformations linked to both health behaviors)																	x	o	o	o	o	o	o	o	o	o	o		
				Advies voor bewegen (education on recommended levels of physical activity)		o	o	o	o	o	o	o	o	o	o	o	o	o	o	o	o	o	o	o	o	o	o	o	o	o	o		
C2.5	C2			Gezond eten (education on healthy diet)		o	o	o	o	o	o	o	o	o	o	o	o	o	o	o	o	o	o	o	o	o	o	o	o	o	o		
				Beter slapen (education on sleep hygiene)		o	o	o	o	o	o	o	o	o	o	o	o	o	o	o	o	o	o	o	o	o	o	o	o	o	o		
				Invloed caffeine en andere middelen (education on impact of caffeine and other substances on both health behaviors)		o	o	o	o	o	o	o	o	o	o	o	o	o	o	o	o	o	o	o	o	o	o	o	o	o	o		
				Omgaan met stress (education on impact of stress on both health behaviors)		o	o	o	o	o	o	o	o	o	o	o	o	o	o	o	o	o	o	o	o	o	o	o	o	o	o		
C3.7	C3			Je lichaam wordt gezonder (education on withdrawal symptoms/muscle soreness being signs for health improvements)																o	o	o	o	o									
Personal motivation																																	
C2.6	C2			Jouw afspraken (commitment to behavioral goals, e.g., by means of a behavioral contract or rule)										o	o	o	o	o	o	o	o	o	o	o	o	o	o	o	o	o	o		
C2.7	C2			Voordelen stoppen met roken en meer bewegen (reasons to quit smoking and increase physical activity, and benefits of both)		o	o	o	o	o	o	o	o	o	o	o	o	o	o	o	o	o	o	o	o	o	o	o	o	o	o	o	
C2.8	C2			Drempels (barriers to stop smoking and increase physical activities and how to overcome them)		o	o	o	o	o	o	o	o	o	o	o	o	o	o	o	o	o	o	o	o	o	o	o	o	o	o	o	
Practical activities																																	
C2.9	C2			Kies jouw sport of beweging (make a plan for physical activity, e.g., look for gym nearby, join a swim club)							x	o	o	o	o	o	o	o	o	o	o	o	o	o	o	o	o	o	o	o	o		
C2.10	C2			Omgeving opnieuw inrichten (environmental restructuring, e.g., throw away your ashtrays, buy running shoes)										o	o	o	o	o	o	o	o	o	o	o	o	o	o	o	o	o	o	o	
Reinforcement																																	
C2.11	C2			Vier je successen (celebrate milestones and progress)										o	o	o	o	o	o	o	o	o	o	o	o	o	o	o	o	o	o		
C2.12	C2			Positief dagboek (keep a positive diary to think about positive things or things you are grateful for)		o	o	o	o	o	o	o	o	o	o	o	o	o	o	o	o	o	o	o	o	o	o	o	o	o	o		
C3.9	C3			Beloning einde-Perfect-Fit-programma (plan to reward yourself once you are done with the PF program)										o	o	o	o	o	o	o	o	o	o	o	o	o	o	o	o	o	o		
