# Supplementary material for: Targeting Key Risk Factors for Cardiovascular Disease in At-Risk Individuals: Developing a Digital, Personalized, and Real-Time Intervention to Facilitate Smoking Cessation and Physical Activity
Source: JMIR Cardio. 2024 Dec 20;8:e47730. doi: 10.2196/47730 (PMC11699499; doi:10.2196/47730)

Multimedia appendix 3.

We developed a refined process that enabled us to work together with a multidisciplinary team on dialogs between user and virtual coach. We give a visual overview of that process here.

In Figure 1, on the following page, a flowchart depicts the different paths a user can take in one of the core dialogs of the virtual coach: the high risk situation (HRS) dialog. The dialog is initiated when the user types 'help'. Depending on the answers of the user the dialog can flow in different directions.

In Figure 2, an excerpt of the Excel sheet we used to draft the conversation is shown. The full excel file has 314 lines. The conversation flows from top to bottom. Column B depicts utterances by the virtual coach, whereas column C holds user responses. The values in column A represent the unique identifier that is used throughout the application's codebase to refer to a set of corresponding utterances in column B. For example `utter_relapse_intro_offer_help` corresponds to the sequence: 'Goed dat je om hulp vraagt!', 'Ik ga je helpen.'. This translates to 'Good that you ask for help!', 'I will help you'. Column D depicts any comments, notice that this often describes the expected dialog flow based on the answer of the user.

The numbered red dashed boxes in Figure 1 and 2 indicate corresponding parts of the figure. Box 1 depicts the initiation of the dialog, asking the user whether they request help related to smoking or physical activity (PA). Box 2 depicts a further specification of what the user needs help with in case of smoking. Similar for box 3, but then for PA (NB: only visible in Figure 1, not in Figure 2). Box 4, 5, & 6 depict different conversations depending in what situation the user is in (HRS, lapse, or relapse).

By making use of the flowchart and Excel sheet, the dialog content and flow could be developed by non-technical team members and this could then be easily picked up by technical members to implement it in the application.

Figure 3 shows screenshots of the conversation as demonstrated in the Excel file (i.e. Figure 2)

Figure 1. Visualization of the structure of the high risk situation dialog collaboratively used by members of the Perfect Fit team to program the dialog in the application.

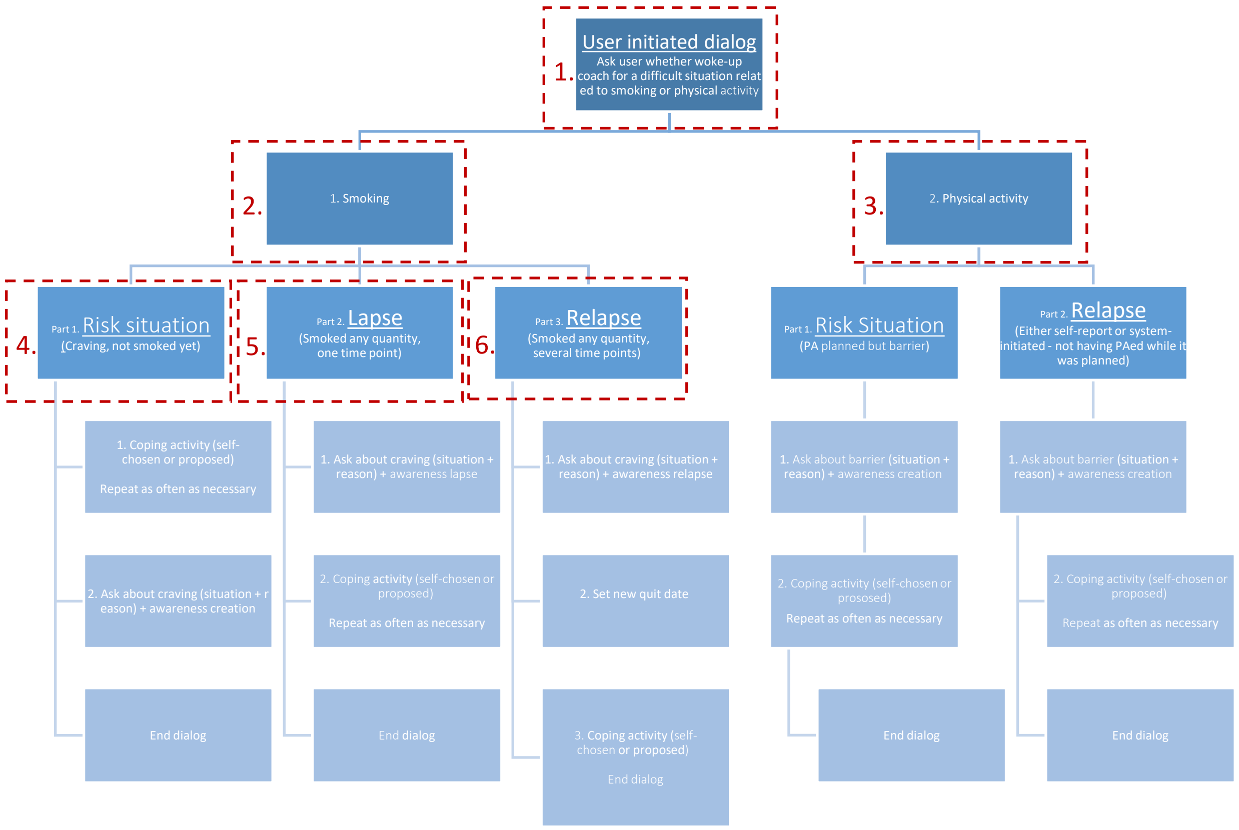

Figure 2. Visualization of the content of the high risk situation dialog collaboratively used by members of the Perfect Fit team to program the dialog in the application.

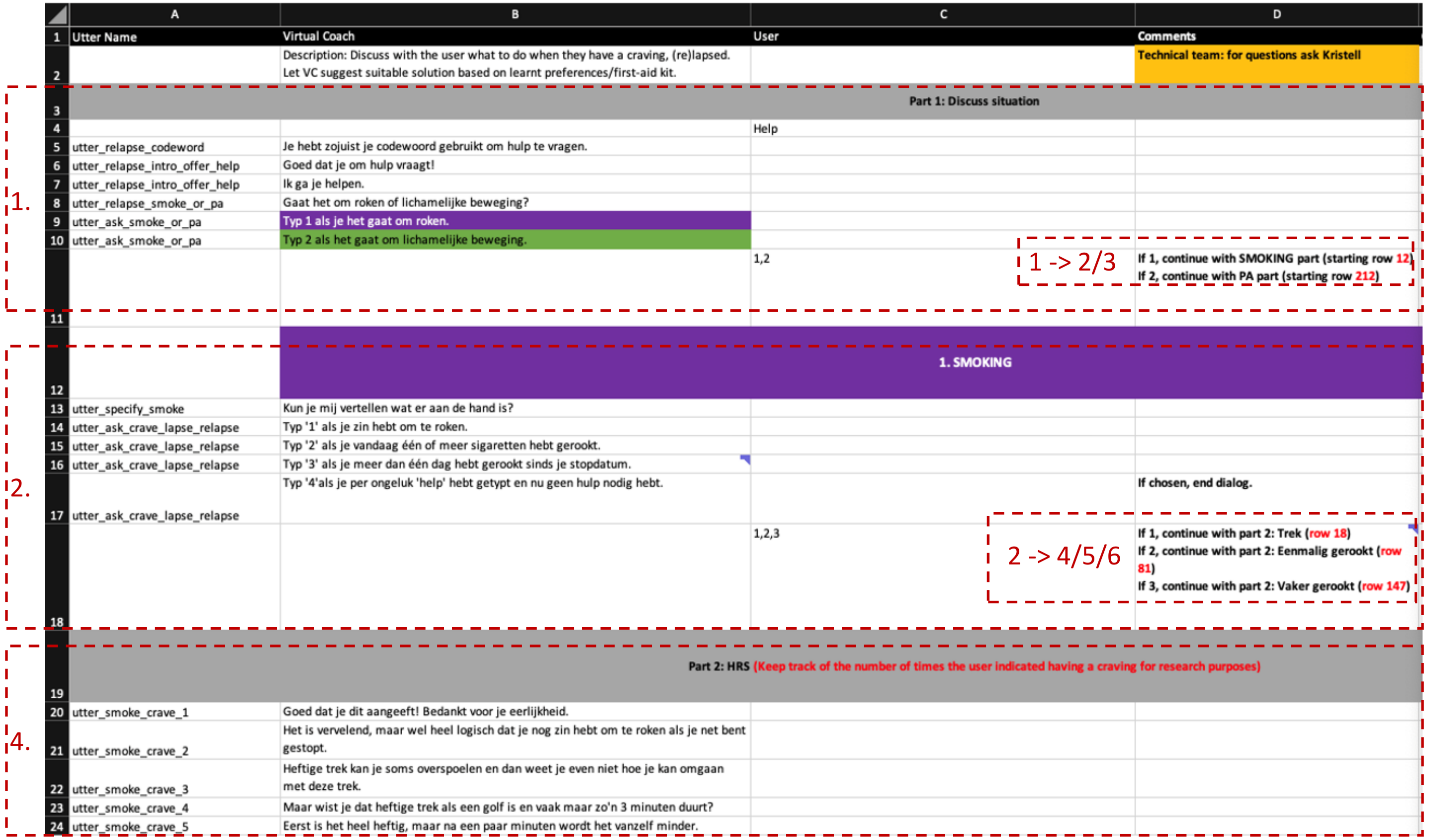

	A	B	C	D
1	Utter Name	Virtual Coach	User	Comments
2		Description: Discuss with the user what to do when they have a craving, (re)lapsed. Let VC suggest suitable solution based on learnt preferences/first-aid kit.		Technical team: for questions ask Kristell
3	Part 1: Discuss situation			
4			Help	
5	utter_relapse_codeword	Je hebt zojuist je codewoord gebruikt om hulp te vragen.		
6	utter_relapse_intro_offer_help	Goed dat je om hulp vraagt!		
7	utter_relapse_intro_offer_help	Ik ga je helpen.		
8	utter_relapse_smoke_or_pa	Gaat het om roken of lichamelijke beweging?		
9	utter_ask_smoke_or_pa	Typ 1 als je het gaat om roken.		
10	utter_ask_smoke_or_pa	Typ 2 als het gaat om lichamelijke beweging.		
11			1,2	If 1, continue with SMOKING part (starting row 12)<br>If 2, continue with PA part (starting row 212)
12		1. SMOKING		
13	utter_specify_smoke	Kun je mij vertellen wat er aan de hand is?		
14	utter_ask_crave_lapse_relapse	Typ '1' als je zin hebt om te roken.		
15	utter_ask_crave_lapse_relapse	Typ '2' als je vandaag één of meer sigaretten hebt gerookt.		
16	utter_ask_crave_lapse_relapse	Typ '3' als je meer dan één dag hebt gerookt sinds je stopdatum.		
17	utter_ask_crave_lapse_relapse	Typ '4'als je per ongeluk 'help' hebt getypt en nu geen hulp nodig hebt.		If chosen, end dialog.
18			1,2,3	If 1, continue with part 2: Trek (row 18)<br>If 2, continue with part 2: Eenmalig gerookt (row 81)<br>If 3, continue with part 2: Vaker gerookt (row 147)
19	Part 2: HRS (Keep track of the number of times the user indicated having a craving for research purposes)			
20	utter_smoke_crave_1	Goed dat je dit aangeeft! Bedankt voor je eerlijkheid.		
21	utter_smoke_crave_2	Het is vervelend, maar wel heel logisch dat je nog zin hebt om te roken als je net bent gestopt.		
22	utter_smoke_crave_3	Heftige trek kan je soms overspoelen en dan weet je even niet hoe je kan omgaan met deze trek.		
23	utter_smoke_crave_4	Maar wist je dat heftige trek als een golf is en vaak maar zo'n 3 minuten duurt?		
24	utter_smoke_crave_5	Eerst is het heel heftig, maar na een paar minuten wordt het vanzelf minder.		

Figure 3. Visualization of the conversation in the application. Both closed ended questions (yes/no, 1/2/3/4) as open questions are supported.

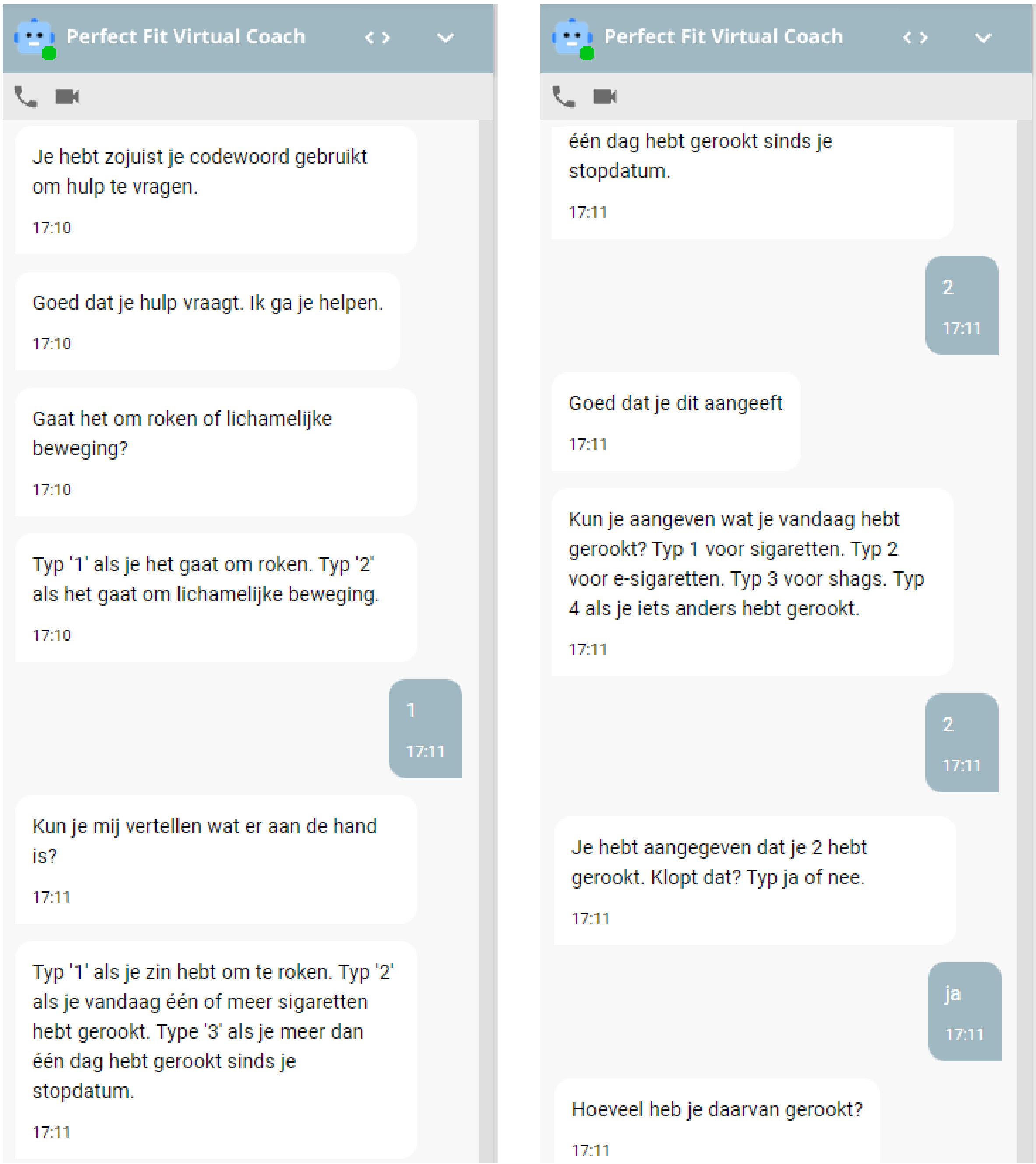

Supplement: Multimedia Appendix 3 [file cardio_v8i1e47730_app3.pdf]
